# Circulating microRNA panels in subjects with metabolic dysfunction-associated steatotic liver disease after following a 2-year dietary intervention

**DOI:** 10.1007/s40618-024-02499-9

**Published:** 2024-11-16

**Authors:** Ana Luz Tobaruela-Resola, José Ignacio Riezu-Boj, Fermín I. Milagro, Paola Mogna-Pelaez, José I. Herrero, Mariana Elorz, Alberto Benito-Boillos, Josep A. Tur, J. Alfredo Martínez, Itziar Abete, María Ángeles Zulet

**Affiliations:** 1 Department of Nutrition, Food Sciences and Physiology, Centre for Nutrition Research, Faculty of Pharmacy and Nutrition, University of Navarra, 31008 Pamplona, Spain; 2 Navarra Institute for Health Research (IdiSNA), 31008 Pamplona, Spain; 3 Centro de Investigación Biomédica en Red de Fisiopatología de la Obesidad y Nutrición (CIBERobn), Instituto de Salud Carlos III, 28029 Madrid, Spain; 4 Liver Unit, Clínica Universidad de Navarra, 31008 Pamplona, Spain; 5 Biomedical Research Centre Network in Hepatic and Digestive Diseases (CIBERehd), 28029 Madrid, Spain; 6 Department of Radiology, Clínica Universidad de Navarra, 31008 Pamplona, Spain; 7 Research group on Community Nutrition and Oxidative Stress, University of Balearic Islands-IUNICS & IDISBA, 07122 Palma, Spain; 8 Precision Nutrition and Cardiovascular Health Program, IMDEA Food, CEI UAM + CSIC, 28049 Madrid, Spain

**Keywords:** MASLD, NAFLD, MiRNA, Biomarker, Nutritional intervention, Fatty liver

## Abstract

**Purpose:**

Metabolic Dysfunction-Associated Steatotic Liver Disease (MASLD) affects one-third of the global population. Despite its high prevalence, there is a lack of minimally non-invasive diagnostic methods to assess this condition. This study explores the potential of circulating microRNAs (miRNAs) as diagnostic biomarkers for MASLD after a 2-year nutritional intervention.

**Methods:**

Fifty-five subjects with steatosis (MASLD group) from the Fatty Liver in Obesity (FLiO) study (NCT03183193) were analyzed at baseline and after 6, 12 and 24 months. Participants were classified into two groups: those who still had steatosis after the intervention (unhealthy group) and those in whom steatosis had disappeared (healthy group). Hepatic status was evaluated through magnetic resonance imaging (MRI), ultrasonography, elastography and serum transaminases. Circulating miRNA levels were measured by RT-PCR.

**Results:**

The dietary intervention was able to modulate the expression of circulating miRNAs after 6, 12, and 24 months. Logistic regression analyses revealed that the most effective panels for diagnosing whether MASLD has disappeared after the nutritional intervention included miR15b-3p, miR126-5p and BMI (AUC 0.68) at 6 months, miR29b-3p, miR122-5p, miR151a-3p and BMI (AUC 0.85) at 12 months and miR21-5p, miR151a-3p and BMI at 24 months (AUC 0.85).

**Conclusions:**

Circulating miRNAs were useful in predicting MASLD in subjects with overweight or obesity after following a weight-loss oriented nutritional intervention. These findings highlight the potential role of miRNAs in diagnosing MASLD and underscore the importance of precision nutrition in managing and determining MASLD.

**Clinical trial registration:**

Trial registration number: NCT03183193 (www.clinicaltrials.gov).

## Introduction

Metabolic dysfunction-associated steatotic liver disease (MASLD), a new terminology proceeded from non-alcoholic fatty liver disease (NAFLD), is closely associated to obesity, diabetes and hypertension [1]. Its prevalence is progressively growing, impacting around one-third of the global population [2].

This new nomenclature, MASLD, includes the requirement of having at least one of five cardiometabolic risk factors [3]. MASLD is characterized by liver steatosis, inflammation or metabolic dysfunction-associated steatohepatitis (MASH), also known as non-alcoholic steatohepatitis (NASH), which represents the most severe manifestation of MASLD [1]. This condition also involves hepatocellular injury and fibrosis, making it the primary liver disorder with a high susceptibility to hepatocellular carcinoma (HCC) [4].

Despite the high prevalence of MASLD and its potential public health impact, there is a lack of effective diagnostic methods for the disease and its advancement to MASH. Liver biopsy, the current gold standard, is invasive and carries risks, highlighting the urgent need for new diagnosis methods to identify MASLD and its evolution, enabling prognosis and guiding treatment [5].

The progression of the disease is markedly influenced by dietary and lifestyle factors. Current clinical guidelines emphasize weight loss as the pivotal component of MASLD management [6], as caloric restriction significantly aids in reducing body weight and various fat deposits [7]. Evidence suggests that energy restriction strategies are viable choices for managing MASLD in individuals with overweight or obesity and adhering to the Mediterranean Diet (MedDiet) may offer additional benefits in weight loss treatments for obesity and its related comorbidities [8].

MiRNAs, which are non-coding RNAs of approximately 22 nucleotides, play crucial roles in liver physiology and pathophysiology [9] as well as in the adipocyte differentiation, fat metabolism regulation, and insulin sensitivity modulation. Alterations in circulating miRNA profiles have been documented in chronic metabolic conditions related to MASLD, such as obesity [10], and are associated with the deregulation of hepatic metabolism, inflammation and liver damage [11]. Environmental and dietary factors have the potential to affect the expression of many miRNAs [12]. Additionally, surgically induced weight loss has demonstrated efficacy in reversing circulating miRNA signature [10]. On the other hand, liver-derived circulating miRNAs show promise as potential biomarkers for diagnosing, monitoring, and predicting MASLD and MASH, and are modifiable by weight loss or insulin-sensitizing treatments [13]. In terms of stability and availability, miRNAs are secreted into body fluids like serum, where they are abundant and relatively stable, making them useful as diagnostic and prognostic indicator for diseases [14].

In this context, considering the impact of weight loss on MASLD and the lack of non-invasive diagnostic methods, our objective was to analyze changes in circulating miRNA levels in subjects with overweight or obesity and MASLD after following a long-term nutritional intervention, and determine whether miRNAs could be used as non-invasive biomarkers for the diagnosis and progression of MASLD.

## Methods

### Study design and participants

The present research is part of the FLiO (Fatty Liver in Obesity) study (NCT03183193), a registered randomized controlled trial. The study protocol was approved by the Ethics Committee of the University of Navarra, Spain, on 24 April 2015 (54/2015), in adherence to the principles outlined in the Declaration of Helsinki. The study was conducted in accordance with the CONSORT 2010 guidelines. All participants provided written informed consent before enrolling in the study.

**Fig. 1:**
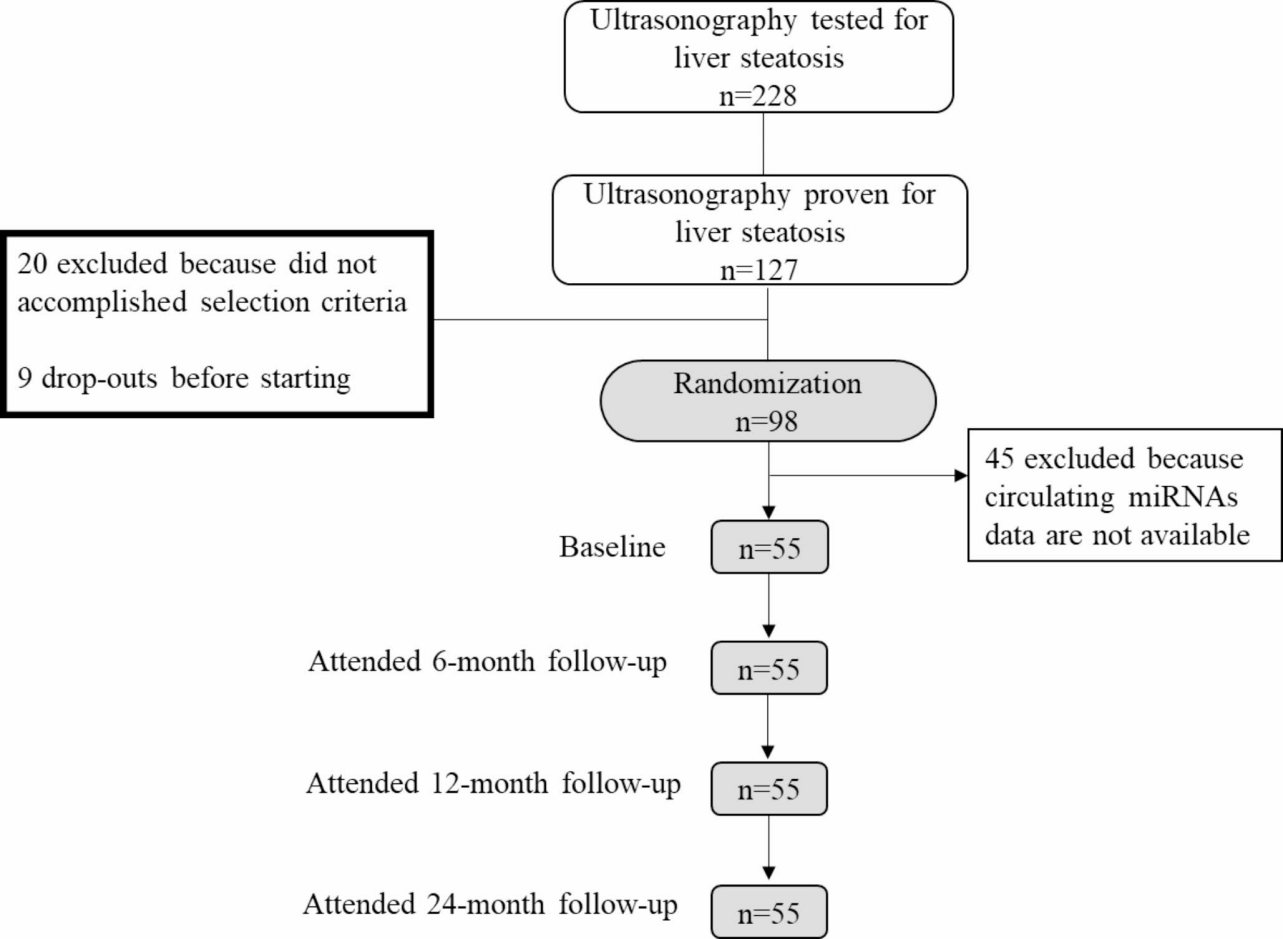
Study Flowchart. *AHA* American Heart Association;* FLiO *Fatty Liver in obesity;* miRNAs* microRNAs

According to the availability of circulating miRNAs data, this study included 55 adults who had overweight or obesity (BMI between 27.5 and 40 kg/m^2^) with MASLD diagnosed by ultrasonography, between 40 and 80 years old. Exclusion criteria were described previously [7].The subjects of the study were randomized into two different dietary strategies (AHA or FLiO), both targeting a significant weight loss of 3–5% over 2 years, which applied a 30% caloric restriction based on total energy requirements (Fig. 1). At the beginning of the study, subjects were randomly assigned to the American Heart Association (AHA) or the Fatty Liver in Obesity (FLiO) groups. Since no differences were found between diets in biochemical, body composition and hepatic variables during the intervention, as demonstrated by Marin-Alejandre et al. [8], both groups were merged into one to enhance the statistical power.

After nutritional intervention, the baseline MASLD group was divided into two subgroups: “unhealthy group”, those who still had MASLD after the nutritional intervention, and “healthy group”, those who no longer suffered from MASLD after each point of the intervention.

Inflammatory markers, hepatic status, biochemical determinations, and body composition were measured in all the subjects at each point of the study. Furthermore, blood samples were collected, processed, and stored at − 80 °C for further analyses [8].

### Lifestyle evaluation

To assess the diet of the subjects at baseline and after 6, 12 and 24 months, a semiquantitative food frequency questionnaire (FFQ) validated in Spain for energy and nutrient intake was used [15]. Each item included in the questionnaire incorporated a standard portion size. The nutrient composition of the food was obtained from recognized Spanish food composition tables. The MedDiet adherence was determined using a 17-point screening questionnaire. Individuals scored between 0 and 17, a higher score indicating a greater adherence to MedDiet [16].

Physical activity (PA) was assessed using the validated Spanish version of the Minnesota Leisure-Time Physical Activity Questionnaire [17]. Energy expenditure in PA was derived under the assumption that 1 MET (Metabolic Equivalent for Task) is equal to 3.5 mL/kg/min [18].

### Body composition and biochemical determination

Body composition, including visceral adipose tissue (VAT), was determined under fasting conditions (8–10 h) in the Nutritional Intervention Unit [8] of the University of Navarra using dual energy X-Ray absorptiometry (Lunar iDXA, enCORE 14.5, Madison, WI) [19]. The Body Mass Index (BMI) was calculated as body weight (BW) divided by squared height (kg/m^2^).

Blood glucose, total cholesterol, high-density lipoprotein (HDL-c), low density lipoprotein cholesterol (LDL-c), triglycerides (TG), alanine aminotransferase (ALT), aspartate aminotransferase (AST) and gamma-glutamyltransferase (GGT) were analyzed on a Pentra C-200 autoanalyzer (HORIBA ABX, Montpellier, France) using precise commercial kits according to the instruction of the manufacturer. LDL-c was calculated with the Friedewald formula, as previously described [20]. The Homeostatic Model Assessment for Insulin Resistance (HOMA-IR) was calculated using the formula elsewhere described [19].

Fatty liver index (FLI) was assessed using an algorithm which included serum TG, BMI, waist circumference, and GGT; a value of ≥ 60 is indicative of hepatic steatosis whereas a value of < 30 indicates absence of it [21]. The FIB-4 (Fibrosis-4) index was calculated as follow: age × AST (U/l)/platelets (10^9^/L) × √ALT (U/l) [22].

### Imaging techniques

Qualified professionals from the Clínica Universidad de Navarra (CUN) performed the hepatic assessment under fasting conditions. MASLD was diagnosed using ultrasonography (Siemens ACUSON S2000 and S3000) following the described methodology [23]. The clinical categorization was performed using a 4-point scale: <5% (grade 0), 5–33% (grade 1), 33–66% (grade 2), > 66% (grade 3) as described previously [24]. Magnetic resonance imaging (MRI) was used to assess liver fat content, volume, and iron, as reported by the manufacturer (Siemens Aera 1.5 T, Erlangen Germany). The quantitative sequences used in the study included the multi-echo T2-corrected single breath-hold spectroscopy (HISTO) [25]. ARFI elastography and transient elastography were carried out to determine liver stiffness as an indirect marker of liver fibrosis [26]. Acoustic radiation force impulse (ARFI) was conducted with the ultrasonography. Transient elastography was performed using FibroScan^®^ (Echosens, Paris, 210 France), as previously described [24]. The median liver stiffness value was estimated by analyzing 10 valid measurements for each subject [27].

### RNA isolation, reverse transcription and real-time (RT-PCR)

Serum was obtained from whole blood through centrifugation at 1100 g at 4 °C for 15 min (Model 5415R, Eppendorf AG, Hamburg, Germany). Subsequently, the samples were frozen at–80 °C until RNA reverse transcription. Total RNA was isolated from serum using the RNeasy Serum/Plasma Advanced Kit (Qiagen), following the manufacturer’s instructions. The procedure involved the use of absolute ethanol (80%) and isopropanol (2-propanol, gradient HPLC grade) (100%) free of nucleases. cDNA was synthesized using 4 µl of miRNA sample and the miRCURY LNA RT Kit (Qiagen, Hilden, Germany). Quantitative PCR (qPCR) was carried out using the CFX384 Touch Real-Time PCR system (Bio-Rad, Hercules, CA, USA) with the miRCURY SYBR^®^ Green PCR Kit and miRCURY LNA miRNA PCR Assays (Ref. 339306, hsa-miR122-5p, hsa-miR-126-5p, hsa-miR-151a-3p, hsa-miR-192-5p, hsa-miR-15b-3p, hsa-miR-29b-3p, hsa-miR-21-5p, hsa-miR-222-3p) (Qiagen, Hilden, Germany). The spike-in UniSp6 was used to assess the quality of the cDNA synthesis (reverse transcription) and qPCR process.

Assays with Cq < 35 were included in the data analysis. The Relative Quantities (RQs) of miRNAs were computed using the formula 2^−ΔCt^, where ΔCt = CtmiRNA − CtUnisp6. The normalization factor (NF) was determined by calculating the geometric mean of RQs for all expressed miRNAs in each sample. The normalized relative quantities (NRQ) were then calculated by dividing RQs by the sample-specific NF, following previously established procedures [28]. The results were expressed as the fold change (FC) for each miRNA concerning the exogenous reference gene, Unisp6.

### Statistical analyses

The normality of the variables was determined with Shapiro-Wilk test. Continuous variables are presented as means and standard deviation (SD) or as median and interquartile ranges (IQR), depending on their distribution. Descriptive statistics were used for comparing data, using Student´s t-test of Mann-Whitney U test according to the data distribution. However, qualitative categorical variables were assessed using the chi-squared test. The differences between various time-points were assessed using paired student`s t-test or Wilcoxon signed-rank test as deemed suitable.

Spearman or Pearson correlations, when appropriate, were performed to explore the associations between hepatic variables and miRNAs (6, 12 and 24 months after intervention) and a heatmap was created. To address the challenge of multiple hypotheses testing, the False Discovery Rate (FDR) was calculated using the Benjamini–Hochberg method [29].

Univariate and multivariate logistic regression analyses were performed with the presence of MASLD after 6,12 and 24 months after intervention as dependent variables. MASLD was categorized into two groups: those who still had mild, moderate or severe steatosis after each point of the intervention (unhealthy group = 1), and those without steatosis (healthy group = 0) [7]. Receiver operating characteristic curve analyses (ROC) and the areas under the ROC curve (AUROC) were determined to evaluate the accurate prediction of miRNAs for MASLD diagnosis. The validation of the results obtained through Tibshirani’s enhanced bootstrap method was performed using an optimism-corrected value as described previously [30].

For multivariate regressions, a statistical command (vselect) was used to choose the best combination of miRNAs to predict MASLD at each point of the study. Then, these combinations were adjusted by sex, age, BMI, PA, and diet groups.

Analyses were performed using Stata version 15.0 (StataCorp 2011, College Station, TX, USA). All reported p-values are two-tailed, and statistical significance was established at *p* < 0.05.

### Generative IA and IA-assisted technologies in the writing process

During the preparation of this work the author used OpenAI to enhance readability and language of the manuscript. After using this tool, the author reviewed and edited the content as needed and took full responsibility for the content of the publication.

## Results

### Effects of dietary intervention on body composition and biochemical parameters

After 6, 12 and 24 months, the healthy group exhibited more pronounced improvements in BMI, VAT, and fat mass compared to the unhealthy group. The healthy group significantly improved the main body composition variables after 6, 12 and 24 months. The unhealthy group showed improvements in all body composition variables after 6 months and in VAT after 12 months (Tables 1, 2 and 3).

The healthy group significantly improved glucose, insulin, HOMA, and TG levels after 6, 12 and 24 months. However, the unhealthy group significantly improved glucose and HOMA after 6 and 12 months. After 6 months of intervention, the healthy group had lower levels of insulin than the unhealthy group. After 12 and 24 months of intervention, the healthy group had lower levels of insulin, HOMA, HDL, and TG than the unhealthy group.

### Impact on physical activity and Mediterranean diet adherence

PA increased significantly after 6, 12 and 24 months of intervention, although there were no significant differences between the unhealthy and the healthy groups (Tables 1, 2 and 3). After 6 months of intervention, there was a significant increase in PA in both groups compared to baseline. However, after 24 months, a significant increase was observed only in the healthy group compared to baseline.

The FFQ analysis revealed a significant increase in the MedDiet adherence score between the healthy and unhealthy groups after 24 months. When analyzing the differences between baseline MASLD group with the subgroups after 6 and 12 months of intervention, a significant decrease in total energy intake, alongside an increase in protein content and MedDiet adherence, were observed in both, healthy and unhealthy groups as well as in the unhealthy group after 24 months. After 6 months of intervention, fiber content significantly increased only in the healthy group. After 12 months, fiber content significantly increased only in the unhealthy group. After 24 months, MedDiet adherence significantly increased only in the healthy group.

### Evaluation of hepatic status

A significant improvement was found in the hepatic status of the healthy group compared with the unhealthy group after 12 months, with ameliorations in steatosis degree, hepatic fat content, liver stiffness, FLI index, and AST as well as after 6 months (Tables 1 and 2). After 24 months, significant improvements were found in steatosis degree, hepatic fat content, FLI index and GGT in healthy group compared to the unhealthy group (Table 3).

After 6 and 12 months of dietary intervention, AST significantly decreased in the healthy group compared to baseline as well as liver volume after 12 and 24 months. FIB-4 increased in the unhealthy group compared to baseline after 6, 12 and 24 months. In addition, significant decreases were found in hepatic fat content, FLI index, ALT, and GGT after 6 and 12 months of intervention in the unhealthy group compared to baseline, with a significant decrease in liver iron noted after 6 months. After 24 months, the healthy group significantly decreased hepatic fat content, TE liver stiffness, FLI index, ALT, and GGT compared to the baseline.

**Table 1:** Body composition, biochemical determinations, lifestyle parameters, inflammatory markers and hepatic status of the MASLD subjects at baseline and after a 6-month nutritional intervention (according to health condition)

	MASLD (*n* = 55)		Healthy group (*n* = 19)	Unhealthy group (*n* = 31)	*P*-value^a^		
	Baseline	6 months	6 months			*P*-value^b^	*P*-value^c^
Body composition, lifestyle and dietary intake							
Weight (kg)	94.7 (14.51)	81.7 (72.9–90.5) ***	78.5 (72.2–86.8)	84.9 (72.9–95.3)	0.342	**< 0.001**	**< 0.01**
BMI (kg/m2)	32.31 (30.22–35.88)	28.43 (27.11–31.64) ***	27.32 (25.7-28.67)	29.60 (27.63–34.38)	**< 0.01**	**< 0.001**	**< 0.01**
VAT (kg)	2.17 (1.60–2.71)	1.41 (0.80–1.85) ***	0.88 (0.70–1.54)	1.42 (1.16–2.09)	**< 0.01**	**< 0.001**	**< 0.01**
Fat mass (kg)	35.87 (32.24–41.52)	28.46 (25.18–33.96) ***	27.12 (18.7-31.57)	32.02 (25.55–35.58)	**0.033**	**< 0.001**	**< 0.001**
PA (METs-min/week)	2238 (1665–4295)	3588 (2400–4740) ***	3240 (2218–5265)	3742 (2400–4185)	0.950	0.057	**0.042**
MedDiet adherence score	6 (4–7)	13 (11–14) ***	13 (12–14)	12 (9–14)	0.169	**< 0.001**	**< 0.001**
Total energy (kcal/day)	2590 (2154–2895)	2066 (518) ***	1952 (523)	2183 (519)	0.157	**< 0.01**	**< 0.01**
Carbohydrates (TEV%)	43.77 (7.01)	41.95 (7.98)	44.31 (5.99)	40.93 (8.94)	0.182	0.780	0.116
Proteins (TEV%)	16.63 (14.60-18.72)	19.19 (16.8.-22.75) ***	20.20 (4.29)	19 (3.54)	0.524	**< 0.01**	**< 0.01**
Lipids (TEV%)	36 (6.93)	36 (8.10)	34 (6.44)	37 (9.25)	0.226	0.141	0.876
Fiber (g/day)	24 (19.67–30.24)	29 (9.05) *	31 (8.61)	28 (9.58)	0.309	**0.025**	0.236
Biochemical determinations							
Glucose (mg/dL)	102 (92–109)	93 (87–99) ***	92 (89–95)	94 (86–102)	0.384	**< 0.01**	**< 0.01**
Insulin (mU/L)	16.3 (12.0-20.9)	8.7 (6.7–13.2) ***	8.1 (5.7–9.1)	9.5 (7.4–13.2)	**0.048**	**< 0.001**	**< 0.001**
HOMA-IR	4.24 (2.94–5.75)	1.95 (1.43–3.19) ***	1.79 (1.25–2.15)	2.29 (1.51–3.38)	0.110	**< 0.001**	**< 0.001**
Total cholesterol (mg/dL)	188.72 (37.07)	176.12 (40.57) **	173.36 (33.73)	176.35 (46.51)	0.809	0.115	0.179
HDL cholesterol (mg/dL)	52.10 (13.97)	54.43 (13.76) *	57.68 (14.12)	52.12 (14.21)	0.185	0.139	0.995
LDL cholesterol (mg/dL)	110.01 (33.13)	101.20 (33.24) *	99.36 (25.47)	101.30 (39.26)	0.850	0.206	0.281
Triglycerides (mg/dL)	123 (86–150)	87 (59–116) ***	72 (56–91)	92 (64–125)	0.118	**< 0.001**	**0.016**
Hepatic status							
Steatosis degree	1 (1–2)	1 (0–1) **	0 (0–0)	1 (1–2)	**< 0.001**	**< 0.001**	0.108
Hepatic fat content (%)	9.2 (5.7–13.9)	5.6 (3.5) ***	3.54 (0.96)	5.5 (4.1–10.6)	**< 0.001**	**< 0.001**	**< 0.01**
Liver vol. (ml)	1697 (1409–2002)	1581 (295) ***	1542 (327)	1592 (290)	0.582	0.072	0.122
Liver iron (%)	26.7 (25.4–29.0)	24.8 (23.1–26.8) ***	24.6 (23-27.6)	25.48 (3.1)	0.810	**0.030**	**< 0.01**
ARFI Liver stiffness (m/s)	1.55 (1.18–2.33)	1.81 (0.67)	1.57 (0.56)	1.96 (0.7)	**0.049**	0.521	0.154
TE Liver stiffness (kPa)	4.4 (3.7–5.9)	4.6 (3.65–5.70)	4.57 (1.01)	4.4 (3.70–6.40)	0.816	0.972	0.868
FLI index	84.5 (73.7–92.3)	48.6 (23.6) ***	34.3 (17.13)	56.3 (23.9)	**0.001**	**< 0.001**	**< 0.001**
ALT (IU/L)	30 (21–43)	21 (16–26) ***	20 (5)	22 (16–28)	0.428	**< 0.001**	**< 0.01**
AST (IU/L)	24 (19–28)	20 (19–25) **	19 (18–21)	23 (19–26)	0.071	**0.011**	0.295
GGT (IU/L)	30 (20–44)	17 (15–26) ***	18 (13–28)	18 (14–27)	0.742	**< 0.01**	**< 0.001**
FIB-4	1.01 (0.79–1.23)	1.24 (0.43) **	1.13 (0.3)	1.31 (0.49)	0.097	0.273	**0.011**

Values are expressed as mean (SD) or median (IQR), according to their distribution. Subjects who still had steatosis after 6 months: unhealthy group. Subjects whom steatosis had disappeared after 6 months: healthy group.

*MASLD* Metabolic Dysfunction-Associated Steatotic Liver Disease; *BMI* Body Mass Index; *VAT* Visceral Adipose Tissue; Med Diet score, Mediterranean Diet Adherence Score;* PA* Physical activity; *HOMA-IR* Homeostatic model Assessment for Insulin Resistance;* HDL* High Density Lipoprotein; *LDL* Low Density Lipoprotein; *ARFI* Acoustic Radiation Force Impulse; *TE* Transient Elastography; *FIB-4 *(Fibrosis-4); *FLI* Fatty Liver Index; *ALT* Alanine aminotransferase; *AST* Aspartate aminotransferase; *GGT* Gamma-glutamyl transferase Significant differences between MASLD at baseline and after 6 months.

*p < 0.05; **p < 0.01; ***p < 0.001. Significant differences between healthy and unhealthy group after 6 months^a^. Significant differences between MASLD at baseline vs. healthy group after 6 months^b^ and between MASLD at baseline vs. unhealthy group after 6 months^c^

**Table 2:** Body composition, biochemical determinations, lifestyle parameters, inflammatory markers and hepatic status of the MASLD subjects at baseline and after a 12-month nutritional intervention (according to health condition)

	MASLD (*n* = 55)		Healthy group (*n* = 22)	Unhealthy group (*n* = 33)	*P*-value^a^		
	Baseline	12 months	12 months			*P*-value^b^	*P*-value^c^
Body composition, lifestyle and dietary intake							
Weight (kg)	94.7 (14.51)	82.8 (76.6–90.0) ***	80.1 (7.1)	84.4 (79.1–99.4)	**0.032**	**< 0.001**	0.067
BMI (kg/m2)	32.31 (30.22–35.88)	29.43 (27.25–32.14) ***	27.24 (2.06)	31.15 (29.24–36.48)	**< 0.001**	**< 0.001**	0.171
VAT (kg)	2.17 (1.60–2.71)	1.57 (0.93–1.87) ***	1.14 (0.52)	1.69 (1.38–2.13)	**< 0.01**	**< 0.001**	**0.047**
Fat mass (kg)	35.87 (32.24–41.52)	29.72 (25.94–37.02) ***	27.58 (22.44–30.25)	33.53 (26.7-38.43)	**< 0.01**	**< 0.001**	0.081
PA (METs-min/week)	2238 (1665–4295)	2818 (1875–5400) *	2611 (1905–5400)	2890 (1620–5547)	1.000	0.204	0.199
MedDiet adherence score	6 (4–7)	11 (9–14) ***	13 (11–15)	11 (8.5–13.5)	0.061	**< 0.001**	**< 0.001**
Total energy (kcal/day)	2590 (2154–2895)	2067.193 (608) ***	1997 (547)	2118 (653)	0.483	**< 0.001**	**< 0.01**
Carbohydrates (TEV%)	43.77 (7.01)	43.41 (7.99)	46.02 (38.62–48.09)	43.68 (8.36)	0.926	0.904	0.957
Proteins (TEV%)	16.63 (14.60-18.72)	19 (17.36–21.38) ***	19 (16.91–21.21)	19 (17.88–21.8)	0.528	**< 0.01**	**< 0.001**
Lipids (TEV%)	36 (6.93)	**34 (5.68)**	35 (4.71)	34 (6.36)	0.629	0.326	0.127
Fiber (g/day)	24 (19.67–30.24)	31 (12.75) **	29 (11.12)	33 (13.84)	0.363	0.054	**< 0.01**
Biochemical determinations							
Glucose (mg/dL)	102 (92–109)	90 (83–101) ***	89.45 (12.12)	92 (84–101)	0.206	**< 0.001**	**< 0.01**
Insulin (mU/L)	16.3 (12.0-20.9)	10.5 (6.8–16.1) ***	9.4 (4.8)	14.7 (8.3–19.2)	**0.010**	**< 0.001**	0.075
HOMA-IR	4.24 (2.94–5.75)	2.36 (1.43–3.81) ***	1.87 (1.24–2.44)	3.29 (1.73–4.07)	**< 0.01**	**< 0.001**	**0.015**
Total cholesterol (mg/dL)	188.72 (37.07)	176.60 (32.70) **	173.40 (25.41)	178.72 (37.00)	0.559	0.080	0.223
HDL cholesterol (mg/dL)	52.10 (13.97)	55.60 (14.10) **	59.13 (12.32)	52.00 (42.00–60.00)	**0.025**	**0.043**	0.890
LDL cholesterol (mg/dL)	110.01 (33.13)	100 (27.09) **	96.13 (20.67)	102.59 (30.67)	0.391	**0.030**	0.298
Triglycerides (mg/dL)	123 (86–150)	91 (70–143) ***	83 (65–106)	109 (74–156)	**0.035**	**< 0.001**	0.315
Hepatic status							
Steatosis degree	1 (1–2)	1 (0–1) **	0 (0–0)	1 (1–2)	**< 0.001**	**< 0.001**	0.642
Hepatic fat content (%)	9.2 (5.7–13.9)	4.1 (3.3–7.4) ***	3.6 (1.4)	5.15 (4.1–10.6)	**< 0.001**	**< 0.001**	**0.013**
Liver vol. (ml)	1697 (1409–2002)	1525 (1341–1736) ***	1535 (250)	1601 (367)	0.476	**0.042**	0.086
Liver iron (%)	26.7 (25.4–29.0)	31.1 (28.7–37.4)	27.5 (4.5)	25.8 (3.2)	0.122	0.544	0.059
ARFI Liver stiffness (m/s)	1.55 (1.18–2.33)	1.72 (1.34–2.3)	1.52 (1.15–1.88)	1.83 (1.47–2.67)	**0.031**	0.547	0.063
TE Liver stiffness (kPa)	4.4 (3.7–5.9)	4.1 (3.50–4.90)	4.10 (3.60–4.60)	4.10 (3.30–5.20)	0.911	0.211	0.294
FLI index	84.5 (73.7–92.3)	53.6 (25.6) ***	35.9 (21.10)	65.3 (21.36)	**< 0.001**	**< 0.001**	**< 0.01**
ALT (IU/L)	30 (21–43)	21 (16–29) ***	20 (9)	23 (19–30)	0.054	**< 0.001**	**0.019**
AST (IU/L)	24 (19–28)	21 (17–25) *	19 (16–22)	22 (19–27)	**0.020**	**< 0.01**	0.422
GGT (IU/L)	30 (20–44)	19 (14–29) ***	19 (13–24)	19 (15–35)	0.530	**< 0.01**	**0.022**
FIB-4	1.01 (0.79–1.23)	1.11 (0.87–1.51)	1.05 (0.83–1.17)	1.18 (0.95–1.61)	0.205	0.973	**0.023**

Values are expressed as mean (SD) or median (IQR), according to their distribution.Subjects who still had steatosis after 12 months: unhealthy group.Subjects whom steatosis had disappeared after 12 months: healthy group.

*MASLD* Metabolic Dysfunction-Associated Steatotic Liver Disease; *BMI* Body Mass Index; *VAT* Visceral Adipose Tissue; Med Diet score, Mediterranean Diet Adherence Score; *PA* Physical activity; *HOMA-IR* Homeostatic model Assessment for Insulin Resistance; *HDL* High Density Lipoprotein;* LDL* Low Density Lipoprotein; *ARFI* Acoustic Radiation Force Impulse; *TE* Transient Elastography;* FIB*-4 (Fibrosis-4);* FLI* Fatty Liver Index; *ALT* Alanine aminotransferase; *AST* Aspartate aminotransferase; *GGT* Gamma-glutamyl ransferase. Significant differences between MASLD at baseline and after 12 months.

*p < 0.05; **p < 0.01; ***p < 0.001. Significant differences between healthy and unhealthy group after 12 months^a^. Significant differences between MASLD at baseline vs. healthy group after 12 months^b^ and between MASLD at baseline vs. unhealthy group after 12 months^c^

**Table 3:** Body composition, biochemical determinations, lifestyle parameters, inflammatory markers and hepatic status of the MASLD subjects at baseline and after a 24-month nutritional intervention (according to health condition)

	MASLD (*n* = 55)		Healthy group (*n* = 26)	Unhealthy group (*n* = 29)	*P*-value^a^		
	Baseline	24 months	24 months			*P*-value^b^	*P*-value^c^
Body composition, lifestyle and dietary intake							
Weight (kg)	94.7 (14.5)	86.9 (77.4–94.5) ***	83.49 (9.8)	93.14 (16.2)	**0.011**	**< 0.001**	0.656
BMI (kg/m2)	32.31 (30.22–35.88)	30.51 (27.48–34.26) ***	28.47 (2.78)	33.85 (4.76)	**< 0.001**	**< 0.001**	0.699
VAT (kg)	2.17 (1.60–2.71)	1.72 (1.27–2.28) ***	1.49 (0.77)	1.88 (1.65–2.56)	**< 0.01**	**< 0.01**	0.859
Fat mass (kg)	35.87 (32.24–41.52)	32.85 (27.34–38.66) ***	30.78 (7.57)	35.96 (28.14–44.85)	**0.010**	**< 0.01**	0.660
PA (METs-min/week)	2238 (1665–4295)	3275 (1632–5107) *	4040 (1878–5275)	2638 (1258–4461)	0.095	**0.025**	**< 0.001**
MedDiet adherence score	6 (4–7)	10 (3) ***	11 (3)	10 (2)	**< 0.01**	**< 0.001**	**< 0.001**
Total energy (kcal/day)	2590 (2154–2895)	2059 (1760–2627) **	2082(1858–2625)	1985 (1586–2658)	0.149	0.055	**< 0.01**
Carbohydrates (TEV%)	43.77 (7.01)	41.42 (7.94)	42.92 (6.76)	40.08 (8.76)	0.196	0.616	**0.044**
Proteins (TEV%)	16.63 (14.60-18.72)	19 (3.54) ***	18 (3.51)	20 (3.44)	0.091	0.131	**< 0.001**
Lipids (TEV%)	36.06 (6.93)	37 (7.44)	36 (6.84)	38 (8.00)	0.473	0.861	0.491
Fiber (g/day)	24.43 (19.67–30.24)	29 (22.97–36.97) **	33 (10.79)	27 (19.87–35.64)	0.129	**0.002**	0.335
Biochemical determinations							
Glucose (mg/dL)	102 (92–109)	92 (85–103) ***	85 (82–89)	97 (92–113)	**< 0.001**	**< 0.001**	0.423
Insulin (mU/L)	16.3 (12.0-20.9)	10.6 (7.1–15.2) ***	6.9 (5.1–10)	14.3 (13.1–18.7)	**< 0.001**	**< 0.001**	0.346
HOMA-IR	4.24 (2.94–5.75)	2.45 (1.41–3.64) ***	1.39 (1.08–2.04)	3.41 (2.99–4.95)	**< 0.001**	**< 0.001**	0.432
Total cholesterol (mg/dL)	188.72 (37.07)	187.83 (41.24)	180.00 (164.00-215.00)	188.00 (1610.0-225.00)	0.309	0.434	0.622
HDL cholesterol (mg/dL)	52.10 (13.97)	52.00 (43.00-62.10)	59.00 (47.00-65.90)	46.50 (40.90–58.00)	**0.016**	0.052	0.451
LDL cholesterol (mg/dL)	110.01 (33.13)	109.18 (32.00)	102.35 (87.50–129.00)	110.95 (84.30-132.50)	0.458	0.575	0.770
Triglycerides (mg/dL)	123 (86–150)	99 (74–162)	77 (61–90)	128 (99–171)	**< 0.001**	**< 0.001**	0.344
Hepatic status							
Steatosis degree	1 (1–2)	1 (0–1) **	0 (0–0)	1 (1–2)	**< 0.001**	**< 0.001**	0.295
Hepatic fat content (%)	9.2 (5.7–13.9)	5.55 (3.5–8.7) ***	3.7 (1.1)	8.7 (6.4–12.8)	**< 0.001**	**< 0.001**	0.964
Liver vol. (ml)	1697 (1409–2002)	1571 (1320–1722) ***	1502 (2461)	1662 (1413–1834)	0.069	**0.011**	0.538
Liver iron (%)	26.7 (25.4–29.0)	26.1 (24.8–28.1) *	25.9 (24.6–26.8)	26.5 (24.9–28.2)	0.315	0.118	0.541
ARFI Liver stiffness (m/s)	1.55 (1.18–2.33)	1.63 (1.26–2.21)	1.53 (1.13–1.91)	1.88 (0.65)	0.142	0.684	0.267
TE Liver stiffness (kPa)	4.4 (3.7–5.9)	3.9 (3.3–5.15)	3.6 (3.1–4.2)	4.45 (1.33)	0.078	**< 0.01**	0.422
FLI index	84.5 (73.7–92.3)	66.8 (41.4–88.6) ***	44.4 (22.5)	88.2 (68.2–91.7)	**< 0.001**	**< 0.001**	0.966
ALT (IU/L)	30 (21–43)	24 (17–29) ***	23.5 (15–29)	24 (20–33)	0.237	**< 0.01**	0.109
AST (IU/L)	24 (19–28)	23 (20–27)	23.5 (21–26)	23 (20–27)	0.872	0.615	0.839
GGT (IU/L)	30 (20–44)	21 (15–29) ***	17 (12–26)	26 (18–31)	**0.027**	**< 0.01**	0.122
FIB-4	1.01 (0.79–1.23)	1.28 (0.45) **	1.26 (0.48)	1.29 (0.43)	0.840	0.062	**0.018**

Values are expressed as mean (SD) or median (IQR), according to their distribution. Subjects who still had steatosis after 24 months: unhealthy group. Subjects whom, steatosis had disappeared after 24 months: healthy group.

*MASLD *Metabolic Dysfunction-Associated Steatotic Liver Disease; *BMI* Body Mass Index; *VAT* Visceral Adipose Tissue; Med Diet score, Mediterranean Diet Adherence Score;* PA* Physical activity; *HOMA-IR *Homeostatic model Assessment for Insulin Resistance; *HDL* High Density Lipoprotein; *LDL* Low Density Lipoprotein; *ARFI *Acoustic Radiation Force Impulse;* TE* Transient Elastography; *FIB*-4 (Fibrosis-4); *FLI* Fatty Liver Index; *ALT* Alanine aminotransferase; *AST* Aspartate aminotransferase;* GGT* Gamma-glutamyl transferase. Significant differences between MASLD at baseline and after 24 months.

*p < 0.05; **p < 0.01; ***p < 0.001. Significant differences between healthy and unhealthy group after 24 months^a^. Significant differences between MASLD at baseline vs. health after 24 months^b^ and between MASLD at baseline vs. unhealthy group after 24 months^c^

### Analyses of circulating miRNA levels and its association with hepatic status

When evaluating circulating miRNA levels, a significant decrease was found after 6 months in miR151a-3p, which was due to the significant decrease observed for the unhealthy group. However, no significant differences were found between the healthy and the unhealthy groups at 6 months (Table 4).

After 12 months of intervention, all miRNAs, except miR192-5p, were significantly downregulated in the unhealthy group compared to the baseline. Moreover, the levels of all the miRNAs, including miR122-5p, were significantly lower in the unhealthy group compared to the healthy group. However, after 24 months of nutritional intervention, miR122-5p levels were significantly lower in the healthy group compared to the unhealthy group.

**Fig. 2:**
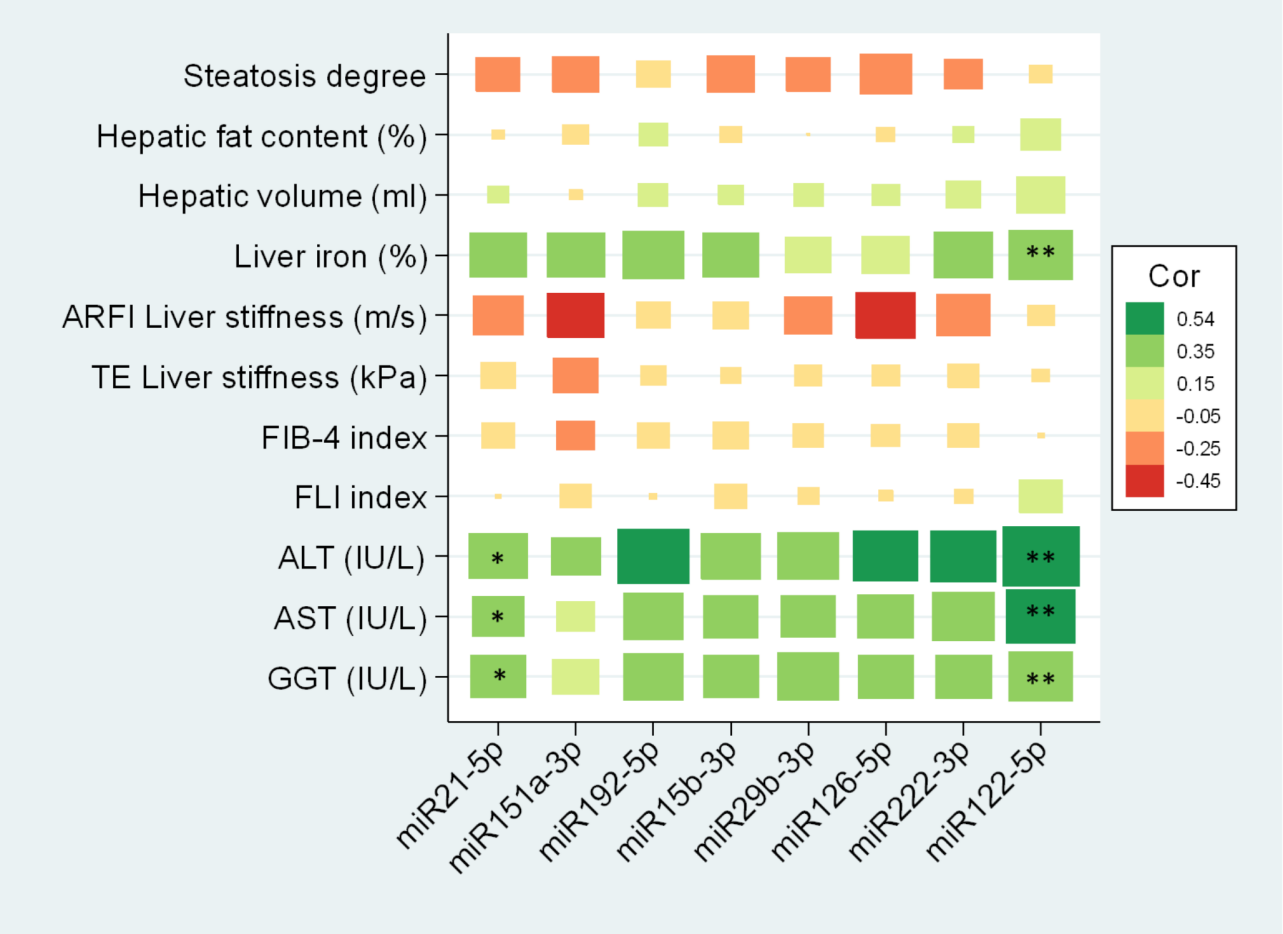
Correlations between miRNAs and hepatic status of the subjects with MASLD after 6-month nutritional intervention. MiRNAs are expressed as Fold Change with respect to UniSp6. Abbreviations: *MASLD* Metabolic Dysfunction-Associated Steatotic Liver Disease; *miR* microRNA; *ARFI *Acoustic Radiation Force Impulse;* TE* Transient Elastography; *FIB*-4 (Fibrosis-4); *FLI *Fatty Liver Index; *ALT* Alanine aminotransferase; *AST* Aspartate aminotransferase; *GGT* Gamma-glutamyl transferase. *p < 0.05; **p < 0.01

**Fig. 3:**
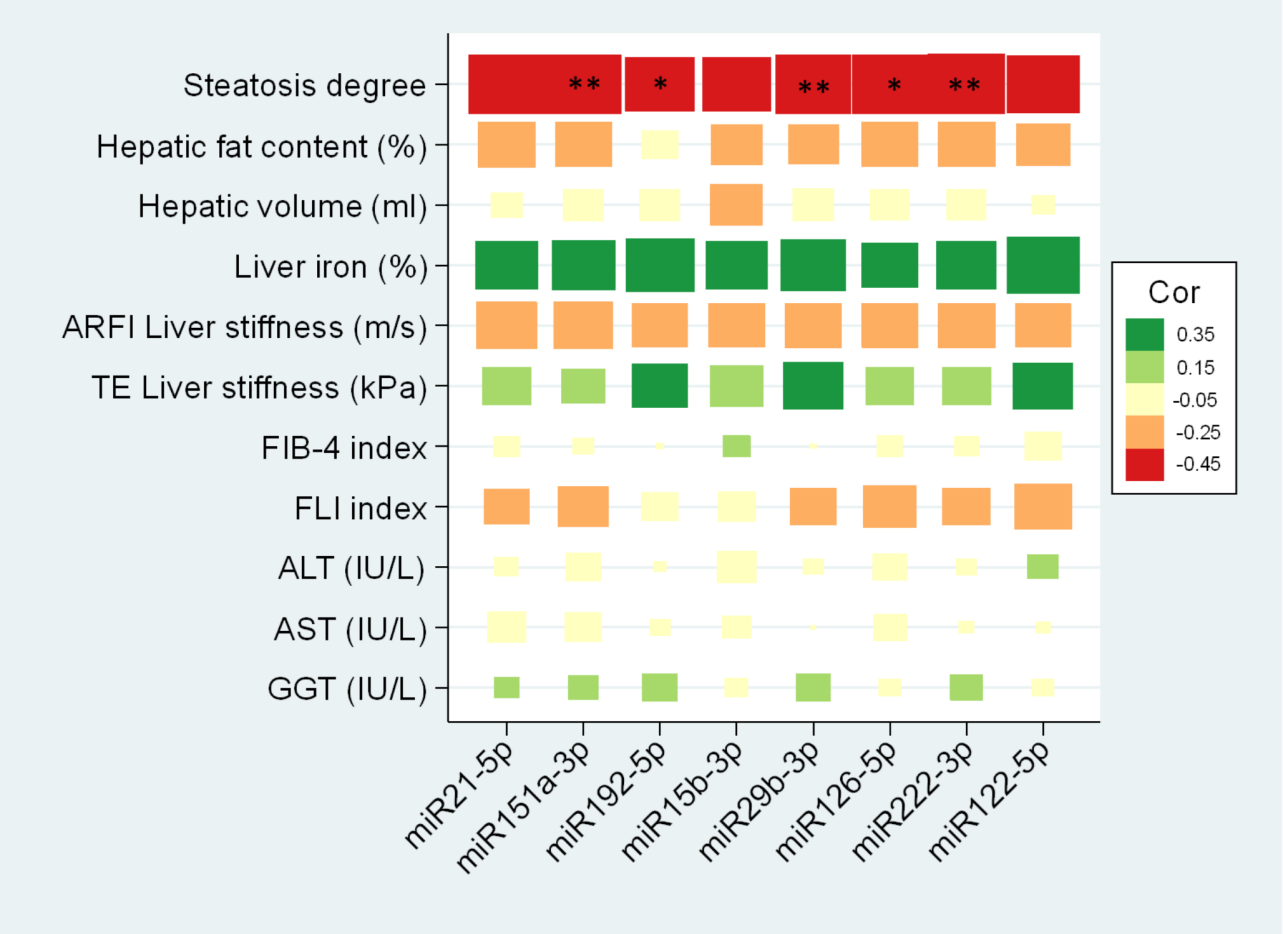
Correlations between miRNAs and hepatic status of the subjects with MASLD after 12-month nutritional intervention. MiRNAs are expressed as Fold Change with respect to UniSp6. Abbreviations: *MASLD *Metabolic Dysfunction-Associated Steatotic Liver Disease; *miR* microRNA; *ARFI* Acoustic Radiation Force Impulse;* TE* Transient Elastography; *FIB*-4 (Fibrosis-4); *FLI* Fatty Liver Index; *ALT* Alanine aminotransferase; *AST* Aspartate aminotransferase; *GGT *Gamma-glutamyl transferase. *p < 0.05; **p < 0.01

The associations between miRNAs and hepatic health variables were further explored. After 6 months of intervention, miR122-5p showed a significant correlation with hepatic iron, ALT, AST and GGT (*p* < 0.01) and miR21-5p with ALT, AST, and GGT (*p* < 0.05) (Fig. 2). Negative associations were found between steatosis degree and miR151a-3p (*p* < 0.01), miR192-5p (*p* < 0.05), miR29b-3p (*p* < 0.01), miR126-5p (*p* < 0.01), and miR222-3p (*P* < 0.05) (Fig. 3) after 12 months of intervention. No significant differences were found at baseline (Fig. S1) and after 24 months of nutritional intervention (Fig. S2).

**Table 4:** Differences between circulating miRNA expression levels of the MASLD subjects at baseline and after a 6-, 12- and 24-month nutritional intervention

miRNAs	MASLD (*n* = 55)		Healthy group (*n* = 19)	Unhealthy group (*n* = 31)	*P*-value^a^		
	Baseline	6 months	6 months			*P*-value^b^	*P*-value^c^
miR21-5p (FC)	1.55 (1.01–2.34)	1.21 (0.63–2.62)	1.41 (0.83–3.31)	1.15 (0.63–2.59)	0.429	0.565	0.298
miR151a-3p (FC)	1.82 (0.93–2.74)	1.26 (0.63–2.03) *	1.42 (0.79–2.54)	1.07 (0.42–1.52)	0.105	0.475	**0.028**
miR29b-3p (FC)	1.8 (0.67–2.73)	1.36 (0.52–2.41)	1.63 (0.84–2.4)	1.02 (0.34–2.24)	0.329	0.832	0.264
miR192-5p (FC)	1.25 (0.79–2.15)	1.35 (0.38–1.93)	1.62 (0.93–1.92)	1.26 (0.35–2.23)	0.430	0.597	0.472
miR222-3p (FC)	1.62 (0.51–2.95)	1.57 (0.56–2.9)	1.65 (0.76–3.19)	1.16 (0.56–2.64)	0.490	0.818	0.555
miR122-5p (FC)	1.57 (0.66–3.46)	1.18 (0.48–2.96)	1.18 (0.58–2.11)	1.56 (0.54–3.07)	0.928	0.428	0.432
miR126-5p (FC)	1.86 (0.75–3.32)	1.5 (0.55–2.54)	1.83 (0.78–3.43)	1.28 (0.54–2.54)	0.276	0.939	0.146
miR15b-3p (FC)	1.49 (0.69–2.39)	1.07 (0.61–2.35)	1.31 (0.87–2.83)	0.83 (0.55–1.83)	0.177	0.893	0.129

miRNAs	MASLD (*n* = 55)		Healthy group (*n* = 22)	Unhealthy group (*n* = 33)	*P*-value^a^		
	Baseline	12 months	12 months			*P*-value^b^	*P*-value^c^
miR21-5p (FC)	1.55 (1.01–2.33)	1.34 (0.46–2.11)	1.93 (1.20)	1.01 (0.39–1.58)	**0.011**	0.340	**0.011**
miR151a-3p (FC)	1.82 (0.92–2.74)	1.19 (0.39–2.27)	2.35 (1.81)	0.9 (0.29–1.36)	**< 0.01**	0.439	**< 0.01**
miR29b-3p (FC)	1.8 (0.66–2.72)	1.43 (0.46–2.22)	2.05 (1.24)	0.7 (0.31–1.81)	**< 0.01**	0.220	**0.022**
miR192-5p (FC**)**	1.25 (0.79–2.14)	1.1 (0.65–2.07)	1.92 (1.27)	0.91 (0.33–1.61)	**< 0.01**	0.097	0.073
miR222-3p (FC**)**	1.62 (0.5–2.94)	1.39 (0.47–2.36)	2.37 (1.5)	0.82 (0.25–1.7)	**< 0.01**	0.157	**0.031**
miR122-5p (FC)	1.57 (0.66–3.46)	1.24 (0.5–2.53)	2.49 (1.74)	0.88 (0.46–1.96)	**0.029**	0.671	**0.021**
miR126-5p (FC)	1.85 (0.74–3.31)	1.6 (0.36–2.8)	2.39 (1.37)	0.98 (0.28–1.94)	**0.011**	0.499	**0.022**
miR15b-3p (FC)	1.48 (0.69–2.38)	1.07 (0.5–2.44)	1.65 (0.93–2.59)	0.91 (0.44–1.41)	**0.024**	0.537	**0.037**

miRNAs	MASLD (*n *= 55)		Healthy group (*n *= 26)	Unhealthy group (*n *= 29)	*P*-value^a^		
	Baseline	24 months	24 months			*P*-value^b^	*P*-value^c^
miR21-5p (FC)	1.55 (1.01–2.33)	1.14 (0.37–3.64)	0.93 (0.26-2)	1.32 (0.37–4.15)	0.363	0.218	0.847
miR151a-3p (FC)	1.82 (0.92–2.74)	0.94 (0.37–3.42)	0.81 (0.41–3.72)	1.12 (0.26–3.09)	0.762	0.516	0.336
miR29b-3p (FC)	1.8 (0.66–2.72)	1.21 (0.53–3.18)	0.96 (0.21–1.78)	1.29 (0.57–3.68)	0.252	0.162	0.987
miR192-5p (FC)	1.25 (0.79–2.14)	1.08 (0.43–3.25)	1 (0.39–1.42)	1.28 (0.46–4.69)	0.218	0.200	0.735
miR222-3p (FC)	1.62 (0.5–2.94)	1.35 (0.45–3.99)	1.11 (0.22–3.43)	1.48 (0.63–4.31)	0.440	0.550	0.915
miR122-5p (FC)	1.57 (0.66–3.46)	0.93 (0.39–3.96)	0.75 (0.23–2.54)	1.14 (0.68–6.03)	**0.039**	**0.048**	0.991
miR126-5p (FC)	1.85 (0.74–3.31)	1.4 (0.44–3.93)	1.45 (0.24–3.92)	1.3 (0.53–3.91)	0.593	0.423	0.732
miR15b-3p (FC)	1.48 (0.69–2.38)	0.99 (0.38–3.29)	0.98 (0.3–2.51)	1.09 (0.38–4.37)	0.373	0.314	0.799

Values are expressed as median and interquartile range (IQR).

*MASLD* Metabolic Dysfunction-Associated Steatotic Liver Disease;* FC* Fold Change; *miR* microRNA. Significant differences were found between MASLD at baseline and after a 6-, 12- and 24-month nutritional intervention,

*p < 0.05. Significant differences between healthy and unhealthy group after 6, 12 and 24 months^a^ after 6, 12 and 24 months. Significant differences between MASLD at baseline vs. health^b^ and between MASLD at baseline vs. unhealthy group^c^ after 6, 12 and 24

### Multivariate logistic regression models

Univariate regression analyses were performed with the presence of MASLD as the dependent variable and miRNAs as the independent variables. MiRNAs were able to predict MASLD only after 12 months of intervention (Table S1). A moderate MASLD-predictive capacity was demonstrated for miR29b-3p (AUC 0.75), miR222-3p (AUC 0.76), miR122-5p (AUC 0.67) and miR126-5p (AUC 0.70). However, no predictive capacity was found for the miRNAs after 6 and 24 months of nutritional intervention.

Next, the best combinations of miRNAs to predict MASLD were selected using a statistical command (vselect) after 6, 12, and 24 months of intervention. Contributing variables such as sex, age, BMI, PA and diet group were included to adjust the models, obtaining multivariate regression analyses.

**Fig. 4:**
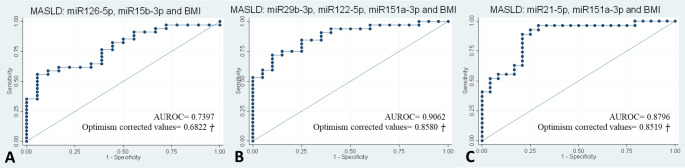
Receiver Operating Characteristic Curves for MASLD with miRNAs and covariables after a **A** 6-, **B** 12- and **C** 24-month nutritional intervention. MiRNAs are expressed as Fold Change with respect to UniSp6. Multivariate models. Model 2: adjusted by BMI. *miR* microRNA; *MASLD *Metabolic Dysfunction-Associated Steatotic Liver Disease;* AUROC *Area under the Receiver Operating Characteristic Curve; *BMI* Body Mass Index

The analyses were performed after 6, 12 and 24 months of nutritional intervention (Table 5). After 6 months, the combination including miR126-5p, miR15b-3p and BMI was able to predict MASLD with an AUC of 0.68 (model 2, Fig. 4).

**Table 5:** Multivariate logistic regressions and ROC curve analyses with MASLD as the dependent factor and miRNAs as the independent variables after a 6-, 12- and 24-month nutritional intervention

Multivariate Models			MASLD (*n* = 55)		
			R2	*P*-value	AUROC
After 6 months of nutritional intervention	Model 1	MiR126-5p (FC), miR15b-3p (FC)	0.0106	0.697	0.5746 (0.5082 †)
	Model 2	MiR126-5p (FC), miR15b-3p (FC) and BMI	**0.1444**	**0.020**	**0.7397 (0.6822 **†**)**
	Model 3	MiR126-5p (FC), miR15b-3p (FC) and PA	0.0116	0.854	0.5490 (0.4336 †)
	Model 4	MiR126-5p (FC), miR15b-3p (FC), Sex, Age and Diet group	0.0255	0.884	0.6063 (0.4577 †)
After 12 months of nutritional intervention	Model 1	MiR29b-3p (FC), miR122-5p (FC), miR151a-3p (FC)	**0.1704**	**< 0.01**	**0.7359 (0.6873 **†)
	Model 2	MiR29b-3p (FC), miR122-5p (FC), miR151a-3p (FC) and BMI	**0.4317**	**< 0.001**	**0.9062 (0.8580 **†)
	Model 3	MiR29b-3p (FC), miR122-5p (FC), miR151a-3p (FC) and PA	**0.1681**	**0.023**	**0.7417 (0.6649 **†)
	Model 4	MiR29b-3p (FC), miR122-5p (FC), miR151a-3p (FC), Sex, Age and Diet group	**0.1981**	**0.032**	**0.7656 (0.6734 **†)
After 24 months of nutritional intervention	Model 1	MiR21-5p (FC), miR151a-3p (FC)	0.0831	0.053	0.6481 (0.6207 †)
	Model 2	MiR21-5p (FC), miR151a-3p (FC) and BMI	**0.3787**	**< 0.001**	**0.8796 (0.8519 **†)
	Model 3	MiR21-5p (FC), miR151a-3p (FC) and PA	0.1012	0.076	0.7090 (0.6517 †)
	Model 4	MiR21-5p (FC), miR151a-3p (FC), Sex, Age and Diet group	0.1267	0.111	0.6991 (0.6112 †)

*miRNA *microRNA; *MASL*D Metabolic Dysfunction-Associated Steatotic Liver Disease;* FC *Fold Change; *AUROC* Area under the Receiver Operating Characteristic Curve; *PA* Physical Activity. † Optimism corrected AUROC value

After 12 months of dietary intervention, all models were able to predict MASLD. The model including only miRNAs (miR29b-3p, miR122-5p and miR151a-3p) showed moderate predictive capacity (AUC 0.68, model 1). When adjusted by PA, the model reached an AUC of 0.66 (model 3) and by sex, age and diet group an AUC of 0.67 (model 4). The combination of the three miRNAs adjusted by BMI, provided the best combination to predict MASLD with an AUC of 0.85 (model 2, Fig. 4).

Finally, the combination including miR21-5, miR151a-3p and BMI, was able to predict MASLD after 24 months of dietary intervention with an AUC of 0.85 (model 2, Fig. 4).

## Discusion

### Therapeutic approaches for MASLD

MASLD is the new nomenclature of NAFLD. The minimal discrepancy between both terms suggests that findings from older NAFLD studies remain valid under MASLD [31].

This study is part of a randomized controlled trial involving 55 subjects with overweight or obesity (BMI > 25) with MASLD, with hepatic steatosis confirmed by ultrasonography. These participants underwent two dietary strategies with a 30% energy restriction for 24 months. This approach is based on previous evidence indicating that a weight loss of 3–5% is recommended to ameliorate hepatic steatosis [32]. Substantial evidence supports the notion that weight loss plays an important role in diminishing pro-inflammatory markers among individuals with obesity or overweight. Additionally, it has been observed that a hypocaloric diet exerts anti-inflammatory effects [33]. In our study, significant improvements in body composition were found after 6, 12, and 24 months of the dietary intervention. Obesity, as estimated by BMI, is highly associated with an elevated risk of MASLD [34]. As BMI increases, MASLD prevalence rises, indicating a direct link between weight gain and liver fat accumulation [8]. This fact aligns with our findings, showing a decrease in hepatic fat content, liver volume, steatosis degree, FLI index, and liver transaminases after weight loss. It has been reported that increased body adiposity plays a key role in circulating miRNA dysregulation [35]. This highlights the importance of dietary habits in the prevention and treatment of MASLD.

### Effect of Mediterranean pattern on MASLD

The MedDiet pattern contributes to improving the severity of MASLD [36]. We observed increased MedDiet adherence after 6, 12 and 24 months of intervention as well as lower energy intake, as has been evidenced to improve hepatic status [32]. Furthermore, our results showed an increase in protein and fiber intake after the intervention. In this context, positive effects on inflammation have been observed after vegetable protein consumption [37]. On the other hand, diets with high fiber content can improve insulin response by decreasing hepatic fat content [38]. Additionally, the fiber derived from fruit has a beneficial impact, specifically improving liver health [39]. These results suggest the importance of maintaining a balanced diet and following a Mediterranean dietary pattern. Another study showed that MASLD subjects after dietary intervention with a MedDiet pattern with olive oil, significantly decreased ALT and AST levels, which are associated with the disease [40], suggesting that this dietary pattern could improve the severity of hepatic steatosis and potentially contribute to improving liver health in subjects with MASLD. In addition to olive oil, the MedDiet also offers a balanced consumption of fruits, vegetables, grains, pulses, fish, and nuts. These foods provide a variety of nutrients known for their anti-inflammatory and antioxidant qualities [41].

### Potential role of circulating miRNAs in MASLD diagnosis

There is a growing focus on examining circulating miRNAs in serum as potential diagnostic biomarkers. Their notable stability and availability make them particularly promising for this purpose [14]. In our research, circulating miRNA levels were measured before, during and after an energy-restricted intervention during 6, 12 and 24 months and a marginally significant decrease was observed after 6 months in miR151a-3p circulating levels. Previous studies have reported a deregulation of circulating miRNA levels in subjects undergoing weight-loss and energy-restricted dietary interventions [42–45].

Alterations in circulating miRNA levels induced by the energy restriction have been reported in subjects with overweight or obesity [10]. After 12 months, we found lower levels of miRNA expression in the unhealthy group compared to the healthy group. This might suggest an adaptive response of the body to the disease as an attempt by the body to counteract liver damage and restore normal liver function and may indicate the active involvement of certain miRNAs in response to the intervention. A previous study reported lower levels of miR126 in subjects with severe MASLD compared to normal or mild MASLD subjects [46]. However, no differences in circulating levels of miR29b were detected between healthy and MASLD subjects in another study [47]. Contrarily, in our study, circulating miR122-5p levels were significantly lower in the healthy compared to the unhealthy group after 24-month intervention. A study proposes that miR122 could be a diagnostic marker for MASLD [48, 49]. This suggests that there are variations in circulating miRNA levels across various physiological stages and pathological conditions.

### Diagnostic accuracy of miRNA combination panels

This study investigated the predictive capacity for MASLD through univariate logistic regression using miRNAs as the only independent variable at different points of the intervention. Specific miRNAs such as miR29b-3p, miR122-5p, miR126-5p, and miR222-3p demonstrated the ability to predict MASLD, particularly after a 12-month intervention. Among human mature miRNAs, miR29b-3p was detectable and significantly upregulated in MASLD subjects [47, 49]. Additionally, miR126 could influence in the development of liver fibrosis [46] and is correlated with improvements in body measurements and glucolipid metabolism after 6-week intervention [50], while positive correlation between changes in miR-126 and changes in BMI were found. On the other hand, miR-222 was found to be linked to adiposity, lipid metabolism, glycemic metabolism, and chronic inflammation. A study showed that MedDiet pattern during the initial stages of pregnancy resulted in a mitigation in the concurrent over-expression of miR222-3p [51]. Others miRNAs analyzed in our study like miR15b, miR192, miR21 and miR151a-3p were not able to predict MASLD effectively in the univariate regression analyses. Contrarily, previous studies showed increased levels of miR15b-3p, miR21-5p, miR29b-3p, miR126-5p, miR151a-3p, and miR192-5p in MASH subjects compared to MASLD subjects [52], and miR122 and miR192 in MASLD or MASH subjects compared to controls [53, 54].

Using a statistical command, miRNA combinations adjusted by BMI were identified for predicting MASLD post-intervention, yielding models showing efficacy after 6, 12, and 24 months. These models incorporate miR126-5p, miR15b-3p and BMI for predicting MASLD after 6 months, miR122-5p, miR151a-3p, miR29b-3p and BMI after 12 months, and miR151a-3p, miR21-5p and BMI after 24 months. Combinations including miR21-5p, miR151a-3p, miR192-5p and miR4449, or miR122, miR192 and miR21, have previously shown satisfactory diagnostic accuracy for MASH in MASLD with AUCs of 0.87 and 0.81, respectively [53, 55]. Another study included miR122-5p, miR1290, miR27b-3p and miR192-5p with a diagnostic accuracy for the disease with an AUC of 0.89 [56]. It can be highlighted that our models include different miRNAs at each time point. The regulation of miRNAs is closely related to dietary habits, particularly the energy and fat content, which are critical factors. Moreover, the duration of the intervention, the selection of samples for analysis, the health status, the method of miRNA detection and the type of dietary strategy employed can affect the miRNA expression [57]. Furthermore, previous studies have shown that polyphenols can regulate miRNA expression both in vivo and in vitro, representing one of the molecular mechanisms underlying their health benefits [58]. The modulation of miRNA expression through dietary interventions, such as extra virgin olive oil (EVOO), may also contribute to improve metabolic outcomes in patients with gestational diabetes mellitus (GDM) [59]. However, while animal models demonstrate metabolic effects, evidence in humans remains limited [57].

### Limitations and strengths of the study

This study has several limitations. Firstly, hepatic status was appraised using non-invasive techniques such as ultrasonography and magnetic resonance imaging, which are convenient but may not provide the diagnostic value of a hepatic biopsy. Secondly, to enhance the technical reliability and consistency among experimental replicates, it is crucial to explore alternative standardized controls in the normalization of miRNAs and, in this study, the exogenous Unisp6 was employed, as suggested by Vigneron et al. [60]. It is noteworthy that not all studies have adopted this reference. Thirdly, as the study focused on participants with overweight or obesity, it is essential to recognize that the conclusions drawn may not be universally applicable to all individuals with obesity. Fourthly, another limitation is sample size and ethnic origin; new large-sample studies not only in Spanish populations are required to support the above findings in the future.

Conversely, the study boasts compelling strengths, notably its randomized controlled design, which is considered the gold standard for assessing dietary interventions. The meticulous participant selection, adhering to stringent inclusion and exclusion criteria, yielded a more homogeneous sample. A distinctive feature is the extended two-year follow-up of participants with MASLD, allowing an exploration of changes in miRNAs, health variables, and behavioral patterns. The mentioned duration is particularly remarkable due to the lack of studies that cover a similar timeframe in this specific field, making it the first study to provide predictive panels of miRNAs for MASLD at different time periods during a 2-year nutritional intervention.

## Conclusion

The combination scores that include miRNAs adjusted by BMI can predict MASLD in subjects with overweight or obesity after 6, 12 and 24 months of dietary interventions. These findings underscore the potential role of circulating miRNAs in diagnosing MASLD and open the door to applying precision nutrition in managing and diagnosing MASLD.

## Electronic supplementary material

Below is the link to the electronic supplementary material.

Supplementary Material 1

## Data Availability

Data available on request due to privacy/ethical restrictions request by contact with the corresponding author (mazulet@unav.es).
