# Supplementary material for: Circulating microRNA panels in subjects with metabolic dysfunction-associated steatotic liver disease after following a 2-year dietary intervention
Source: J Endocrinol Invest. 2024 Nov 16;48(4):987–1003. doi: 10.1007/s40618-024-02499-9 (PMC11950055; doi:10.1007/s40618-024-02499-9)
Supplement: Supplementary file 1 — Supplementary Material 1 [file 40618_2024_2499_MOESM1_ESM.docx]

**Supplementary Information**

**Circulating microRNA panels in subjects with Metabolic Dysfunction-Associated Steatotic Liver Disease after following a 2-year dietary intervention**

J Endocrinol Invest

Ana Luz Tobaruela-Resola^1^, José Ignacio Riezu-Boj^1,2^, Fermín I. Milagro^1,2,3^, Paola Mogna-Pelaez^1^, José I. Herrero^2,4,5^, Mariana Elorz^2,6,^ Alberto Benito-Boillos^2,6^, Josep A. Tur ^3,7^, J. Alfredo Martínez^3,8^, Itziar Abete^1,2,3#^, M. Ángeles Zulet^1,2,3#*^.

^1^Department of Nutrition, Food Sciences and Physiology and Centre for Nutrition Research, Faculty of Pharmacy and Nutrition, University of Navarra, 31008 Pamplona, Spain.

^2^Navarra Institute for Health Research (IdiSNA), 31008 Pamplona, Spain.

^3^ Centro de Investigación Biomédica en Red de Fisiopatología de la Obesidad y Nutrición (CIBERobn), Instituto de Salud Carlos III, 28029 Madrid, Spain.

^4^Liver Unit, Clínica Universidad de Navarra, 31008 Pamplona, Spain.

^5^Biomedical Research Centre Network in Hepatic and Digestive Diseases (CIBERehd), 28029 Madrid, Spain

^6^Department of Radiology, Clínica Universidad de Navarra, 31008 Pamplona, Spain.

^7^Research group on Community Nutrition and Oxidative Stress, University of Balearic Islands-IUNICS & IDISBA,07122 Palma, Spain.

^8^Precision Nutrition and Cardiovascular Health Program, IMDEA Food, CEI UAM + CSIC, 28049 Madrid Spain.

#Contributed equally

*Authors to whom correspondence should be addressed.

mazulet@unav.es

**Supplementary Fig. 1** Correlations between miRNAs and hepatic status of subjects with MASLD at baseline. MiRNAs are expressed as Fold Change with respect to UniSp6. Abbreviations: MASLD, Metabolic Dysfunction-Associated Steatotic Liver Disease; miR, microRNA; ARFI; Acoustic Radiation Force Impulse; TE, Transient Elastography; FIB-4, Fibrosis-4; FLI, Fatty Liver Index; ALT, Alanine aminotransferase; AST, Aspartate aminotransferase; GGT, Gamma-glutamyl transferase.

**Supplementary Fig. 2** Correlations between miRNAs and hepatic status of the subjects with MASLD after 24-month nutritional intervention. MiRNAs are expressed as Fold Change with respect to UniSp6. Abbreviations: MASLD, Metabolic Dysfunction-Associated Steatotic Liver Disease; miR, microRNA; ARFI; Acoustic Radiation Force Impulse; TE, Transient Elastography; FIB-4, Fibrosis-4; FLI, Fatty Liver Index; ALT, Alanine aminotransferase; AST, Aspartate aminotransferase; GGT, Gamma-glutamyl transferase.

**Supplementary Table 1**. Univariate logistic regressions and ROC curve analyses between MASLD as the dependent factors and miRNAs as predictive factors.

| Univariate model | MASLD (*n*=55) | | | | | | | | |
| --- | --- | --- | --- | --- | --- | --- | --- | --- | --- |
|  | **After 6 months of intervention** | | | **After 12 months of intervention** | | | **After 24 months of intervention** | | |
|  | **β** | **P-value** | **AUROC** | **β** | **P-value** | **AUROC** | **β** | **P-value** | **AUROC** |
| miR21-5p (FC) | 0.907 | 0.614 | 0.5671(0.5043 ⴕ) | 0.704 | 0.134 | 0.7039 (0.6949 ⴕ) | 1.170 | 0.216 | 0.5729 (0.5465 ⴕ) |
| miR151a-3p (FC) | 0.926 | 0.686 | 0.6398 (0.5933 ⴕ) | 0 .729 | 0.094 | 0.7396 (0.7352 ⴕ) | 1.013 | 0.858 | 0.4753 (0.4332 ⴕ) |
| miR29b-3p (FC) | 1.005 | 0.970 | 0.4158 (0.3639 ⴕ) | 0.654 | **0.038** | 0.7545 (0.7546 ⴕ) | 1.184 | 0.186 | 0.5965 (0.5718 ⴕ) |
| miR192-5p (FC) | 0.909 | 0.664 | 0.5681 (0.5184 ⴕ) | 0.699 | 0.097 | 0.7273 (0.7165 ⴕ) | 1.182 | 0.159 | 0.5986 (0.5802 ⴕ) |
| miR222-3p (FC) | 0.963 | 0.832 | 0.5586 (0.5021 ⴕ) | 0.497 | **0.005** | 0.7667 (0.7673 ⴕ) | 1.100 | 0.367 | 0.5625 (0.5284 ⴕ) |
| miR122-5p (FC) | 0.992 | 0.957 | 0.4924 (0.4340 ⴕ) | 0.588 | **0.015** | 0.6749 (0.6766 ⴕ) | 1.126 | 0.231 | 0.6717 (0.6428 ⴕ) |
| miR126-5p (FC) | 0.951 | 0.728 | 0.5925 (0.5354 ⴕ) | 0.557 | **0.008** | 0.7039 (0.7088 ⴕ) | 1.100 | 0.328 | 0.5429 (0.5112 ⴕ) |
| miR15b-3p (FC) | 0.961 | 0.771 | 0.6165 (0.5745 ⴕ) | 0.928 | 0.557 | 0.6887 (0.6587 ⴕ) | 1.213 | 0.141 | 0.5752 (0.5693 ⴕ) |

Abbreviations: miR, microRNA; MASLD, Metabolic Dysfunction-Associated Steatotic Liver Disease; FC, Fold Change; AUROC, Area under the Receiver Operating Characteristic Curve. ⴕ Optimism corrected AUROC value.
